# Tool Wear State Identification Method with Variable Cutting Parameters Based on Multi-Source Unsupervised Domain Adaptation

**DOI:** 10.3390/s25061742

**Published:** 2025-03-11

**Authors:** Zhigang Cai, Wangyang Li, Jianxin Song, Hongyu Jin, Hongya Fu

**Affiliations:** 1 School of Mechatronic Engineering, Harbin Institute of Technology, Harbin 150001, China; zhgcai@hit.edu.cn (Z.C.); song22193625@163.com (J.S.); hongyafu@hit.edu.cn (H.F.); 2 Inspur Genersoft Co., Ltd., Jinan 250101, China; leocooki@163.com

**Keywords:** tool wear state identification, transfer learning, multi-source unsupervised domain adaption, varying cutting parameters

## Abstract

Accurately identifying tool wear states with variable cutting parameters can improve machining quality and efficiency. However, existing wear state recognition methods based on unsupervised domain adaptation mostly employ the knowledge transfer learning strategy in a single source domain. They cannot fully utilize the sensor data distribution information of multiple cutting parameters, hindering recognition performance improvement. Thus, this paper proposes a wear-state recognition method for variable cutting parameters based on multi-source unsupervised domain adaptation. First, non-stationary Transformer encoders extract non-stationary common features; then, sliced Wasserstein distance-based domain-specific feature distribution alignment and classifier output alignment scale down the domain shift and make multi-domain distribution synchronous alignment less complex. Finally, the milling experiments with variable cutting parameters are conducted to validate the recognition performance of the proposed method.

## 1. Introduction

Tool wear is one of the critical factors affecting machining quality and machining efficiency [1]. Severely worn tools cause the surface quality of the workpiece to deteriorate and reduce the dimensional accuracy of the workpiece. If processing continues until the tool is damaged, the workpiece will be scrapped, and even the machine tool will be damaged. According to statistics, tool wear or breakage accounts for about 20% of downtime and economic losses [2]. However, tool replacement relies on manual experience to avoid excessive wear, resulting in only 50–80% of the practical tool life being used [3]. Therefore, accurately predicting tool wear state is significant for improving machining efficiency. Existing tool wear state prediction models can be divided into physics-based and data-driven models [4]. Physics-based models are mathematical models built based on knowledge of physical models, laws, and measurement data, and their performance depends mainly on the quality and accuracy of the knowledge in the relevant field. The inability to update the model using online monitoring data limits the effectiveness and flexibility of the physics-based models. The data-driven model uses historical monitoring data for modeling and attempts to update the model and make decisions based on new online monitoring data. Thanks to the widespread application of intelligent sensing and the rapid development of machine learning technology, data-driven tool wear prediction research has become a hotspot in recent years.

With the rapid development of deep learning and computing power, deep learning has attracted extensive attention in academia and industry as it demonstrates automatic feature extraction and great representation learning capabilities for data based on deep network structures [5,6]. Common deep networks for wear state identification include autoencoders (AE) [7], convolutional neural networks (CNN) [8,9], recurrent neural networks (RNN) [10], Transformers, etc. Additionally, Yu et al. [11] proposed Pareto-optimal Adaptive Loss Residual Shrinkage Network (PALRSN), which improves the recognition accuracy of small sample categories through the adaptive loss function. Li et al. [12] proposed a tool wear prediction method based on Informer encoder and a stacked bidirectional gated recurrent unit. In general, these studies have achieved good performance by utilizing deep networks for tool wear detection research. However, in practical processing scenarios, collecting large amounts of labeled data for training is highly labor-intensive and costly. Additionally, changes in workpieces, cutting tools, and cutting parameters can lead to variations in cutting conditions within the machining scenario [12]. This affects the data distribution used for model training and machining monitoring, resulting in a significant decline in the recognition performance of the aforementioned models.

To address the problem of wear state identification under variable cutting conditions, many scholars have introduced the transfer learning paradigm into monitoring model development to reduce the amount of data required. Transfer learning-based wear state identification methods generally aim to learn transferable common knowledge from historical cutting conditions containing rich data (source domain) and utilize it in target cutting conditions with sparsely labeled data or unlabeled data (target domain). Based on the data labeling of the source and target domains, Pan et al. [13] categorized the transfer learning tasks into inductive transfer learning, transductive transfer learning, and unsupervised transfer learning. According to the research, there are two main types of transfer task scenarios for tool wear state identification under variable working conditions, i.e., inductive transfer learning where the source domain consists of labeled data, and domain adaption (DA).

In the inductive transfer learning task scenario, researchers often apply the transferred knowledge to the wear state identification under target cutting conditions based on feature transfer and parameter/model transfer methods. For the feature transfer-based approach, they attempt to seek common features in the two domains by analyzing the feature correlations between the source and target domains to achieve the transfer of tool wear knowledge. For example, Li et al. [14] used a genetic algorithm to generate candidate feature subsets in the source and target domains, and then transferred the feature information from the source domain to the target domain through a relational model and obtained the optimal feature subset based on the maximum mean discrepancy (MMD) metric, and finally realized the tool wear state identification in the target domain based on the particle swarm-optimized support vector machine. For parameter/model transfer-based wear state identification methods, they assume that several network model parameters or hyper-parameters are shared between the source domain task and the target domain task so that the model can be transferred to the identification task under the new working condition through pre-training and fine-tuning [15], etc. For instance, Zhang et al. [16] constructed a wear state identification model based on the model transfer method. They realized the tool wear state identification under variable feed rate by freezing the shallow feature extractor of the pre-trained improved deep residual network and fine-tuning the high-level feature extractor and classification network with cutting force signals as input after wavelet transform. Bahador et al. [17] also realized the tool wear state recognition under different machining equipment based on the pre-training and fine-tuning approach by freezing the CNN feature extractor and fine-tuning the training of the fully-connected classification network using the data from the target domain.

DA-based, especially unsupervised domain adaptation (UDA)-based [18,19], wear state identification methods relax the labeled data requirement for the target domain, which can only utilize the data under known cutting conditions and the unlabeled data under the new cutting conditions for transfer knowledge learning, which reduces the monitoring task cost to a certain extent. The UDA methods commonly used for wear state identification under variable cutting conditions can be divided into two categories: discrepancy-based methods [20,21] and adversarial-based methods [22,23]. Adversarial-based methods achieve the learning of domain-invariant features by designing the domain discriminator or adversarial objectives during the training process to encourage domain confusion. For instance, Li et al. [24] proposed a dynamic domain adversarial self-adaptive method for tool wear state recognition under different milling conditions. Discrepancy-based methods measure the discrepancies between the source and target domains within a model-specific network layer, e.g., using statistical metrics, and thus seek alignment between the source and target domains. For example, Liu et al. [25] proposed an interpretable domain adaptation Transformer for the transferable fault diagnosis. This method utilizes a multi-layer domain adaptation Transformer framework to capture key global information for learning domain modulation information, while minimizing feature distribution discrepancies.

The above wear state identification methods under variable cutting conditions for unlabeled target domains are mostly used to establish a monitoring task transfer between a known cutting condition and a new condition; however, in practice, there are monitoring data corresponding to more than one cutting condition. For the wear state monitoring in this case, a natural way to deal with it is to integrate the historical data from multiple conditions into one source domain. For example, Kim et al. [26] proposed a multi-domain mixture density network, which maps multi-sensor data from multiple cutting conditions to a common feature space and combines it with an adversarial learning method to guide the model to learn the public domain invariant representation. Zhu et al. [27] also proposed an unsupervised dual-regression domain adversarial adaptation network, which integrates data from multiple machining conditions into a single source domain and utilizes the weight discrepancy restriction and prediction consistency loss to align the distributions between domains, and then realizes the prediction of tool wear value. However, the distribution of monitoring data is different among different cutting conditions, and directly integrating multi-condition data into a single-source domain for single-source unsupervised domain adaptation (SUDA) [28] is prone to ignoring the feature distributions among different conditions during the process of learning domain invariant representation, resulting in a negative transfer effect.

Based on the above research status, we proposed a tool wear state identification method with variable cutting parameters based on multi-source unsupervised domain adaptation (MUDA). Overall, this method treats monitoring data in multiple cutting parameters as separate source domains and jointly uses unlabeled data in target cutting parameters as input to build a cross-domain wear state identification model. This model can identify the difference in feature distributions between multiple known cutting parameters and target cutting parameters, automatically extract high-level domain-specific invariant representations, and then achieves effective identification of the wear state under target cutting parameters, with an average accuracy exceeding 90%. The main contributions of this study are as follows.

(1) A novel multi-source domain adaptive tool wear state prediction method based on Multiple Feature Spaces Adaptation Network (MFSAN) architecture is proposed. This method achieves tool wear state prediction under varying cutting parameters by constructing a multi-feature space adaptation network.
(2) A public feature extractor based on a Non-Stationary Transformer Encoder (NSTE) is proposed. This extractor utilizes a sequence stationarization module and NSTE to explore non-stationary input features in multi-channel signals, thereby extracting advanced public features related to wear.
(3) The proposed model incorporates a domain-specific feature distribution alignment module based on sliced Wasserstein distance (SWD) and a domain-specific classifier output alignment module. SWD allows for the measurement of differences in the hidden feature space with low computational cost. These two alignment modules mitigate domain shift and simplify the synchronization of alignment across multiple domain distributions.

## 2. Proposed Method

### 2.1. Problem Description

This paper investigates tool wear state monitoring under variable cutting parameters based on MUDA, aiming to construct an effective cross-domain wear state identification model with data under multiple existing cutting parameters. The proposed model can identify the feature distribution discrepancies between multiple existing cutting parameters and the target cutting parameters and extract high-level domain-invariant representations, directly realizing the accurate identification of wear states under the target cutting parameters.

Several basic assumptions and formulations are made to depict the problem. First, multi-channel sensor history data exist for multiple existing cutting parameters, and the variation in cutting parameters such as cutting speed, spindle speed, and depth of cut leads to large differences in the distribution of sensor data collected under different cutting parameters. The sensor data under a single cutting parameter and its wear state can form a domain *D*, formally described as $D=\left\{{\left(x_{l},y_{l}\right)}_{l=1}^{L}\right\}$. Among them, $x_{l}$ is the input generated by multi-channel sensor data in domain *D*, $y_{l}$ is the corresponding wear state label, there are *C* label values, and *L* is the number of samples in domain *D*. Sensor data and wear state under multiple existing cutting parameters form multiple domains, which are the source domain $D^{S}$ in unsupervised domain adaptation. There are *M* source domains $D_{i}^{S}=\left\{{\left(x_{i,l},y_{i,l}\right)}_{l=1}^{L_{i}}\right\},1\leq i\leq M$ in $D^{S}$ and enough labeled samples in each source domain to build an effective cross-domain classifier. The sensor data collected under the target cutting parameters form the target domain $D^{T}$, and the single target domain is $D^{T}=\left\{{\left(u_{k}\right)}_{k=1}^{K}\right\}$. There are only *K* small numbers of unlabeled samples $u_{k}$ in the target domain, and an effective wear state recognition model cannot be constructed using only these unlabeled samples. In addition, the wear state label spaces of each source domain and target domain mentioned above are identical, but the marginal distribution of each domain is noticeably distinct.

**Figure 1:**
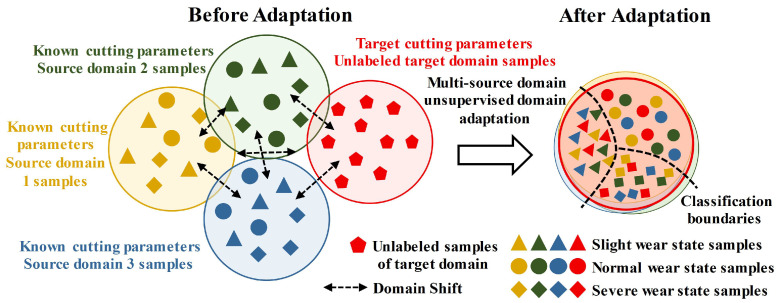
Schematic diagram of tool wear state identification based on multi-source unsupervised domain adaptation.

As depicted in Figure 1, MUDA can effectively learn from many labeled samples in the presence of existing cutting parameters and a limited number of unlabeled target domain samples, in contrast to SUDA. MUDA utilizes a cross-domain high-level feature extractor *F* and classifier *T* to decrease the domain shift resulting from differences in marginal distribution across various cutting parameters. This method reduces the reliance on labeled samples from the target domain when training the model. It also enables the efficient transfer of wear knowledge from multiple source domains to the target domain, creating a precise classification boundary for tool wear state in the target domain.

### 2.2. The Method for Tool Wear State Recognition Based on MFSAN

In order to solve the problem existing in unsupervised domain adaptation, Zhu et al. proposed the MFSAN, which can align domain-specific distributions and domain-specific classifiers in two stages [29]. MFSAN can serve as a generalized multi-source unsupervised domain adaptive architecture. On the one hand, it maps the target domain and each source domain to different feature spaces separately. It performs domain-specific distribution alignment to learn multiple domain-invariant representations, which reduces the difficulty of acquiring domain-invariant representations while entirely using multi-source domain samples for feature learning. On the other hand, MFSAN considers the relationship between the target domain samples and the domain-specific classification boundary and uses the domain-specific decision boundary to align the unlabeled target domain samples through the classifier output, improving the classification ability on the target domain.

**Figure 2:**
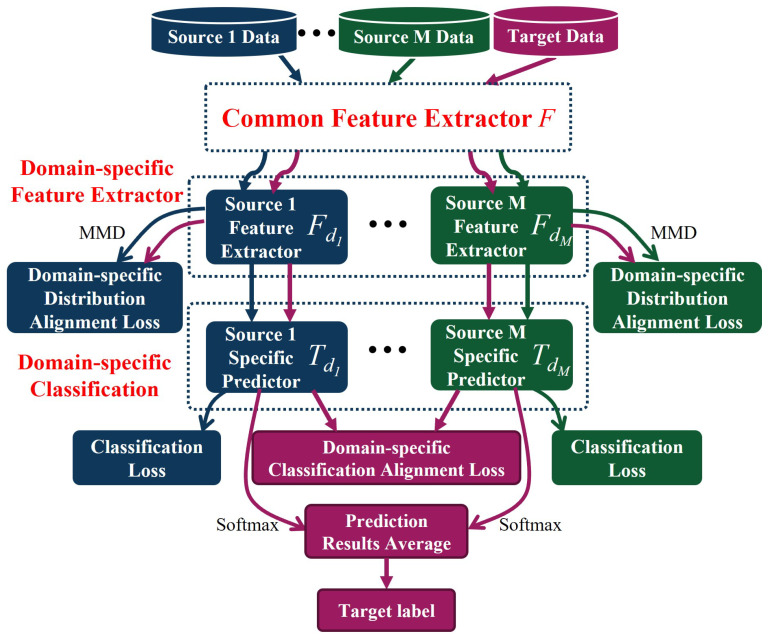
Multiple feature space adaptation network based on two-stage alignment.

Figure 2 illustrates the overall network architecture of MFSAN. In order to reduce the complexity of the network structure, the feature extractor *F* consists of a common feature extractor $F_{1}$ that shares parameters and multiple domain-specific feature extractors $F_{d}={\left\{F_{d_{i}}\right\}}_{i=1}^{M}$. The classifier is a multi-output network with predictors $T_{d}={\left\{T_{d_{i}}\right\}}_{i=1}^{M}$ corresponding to each domain-specific feature extractor. All source domain and target domain data first enter the common feature extractor $F_{1}$ to extract the common representations of all domains. In order to map each pair of source and target domains into a domain-specific feature space, MFSAN designs a domain-specific extractor $F_{d_{i}}$ for each source domain. The network parameters are not shared among the domain-specific feature extractors, so domain-specific domain-invariant representations between multiple source and target domains can be obtained. During training, the differences between individual source and target domains can be minimized in various approaches, such as statistical difference measure loss, adversarial loss, etc. A single domain-specific predictor $T_{d_{i}}$ outputs the corresponding predicted labels through a Softmax classifier after receiving the high-level features of the corresponding source domains.

There are two alignment stages in the MFSAN architecture: the alignment of domain-specific distributions and the alignment of domain-specific classifiers. In the domain-specific distribution alignment stage, the respective high-level features of each pair of source and target domains are obtained by mapping the respective domain-specific feature networks. The MMD calculates the discrepancy in feature distribution between the source and target domains for each pair, which is then utilized as one of the loss functions. Minimizing this loss function motivates each domain-specific extractor to learn a domain-invariant representation of the source and target domains for each pair. Since the target domain samples are trained on different predictors, the output of each classifier on the target sample diverges. In particular, samples close to the classification boundary are more likely to be misclassified. Therefore, in the domain-specific classifier alignment stage, MFSAN uses the absolute value of the mutual difference between the probability outputs of the target domain samples on all predictors as the difference loss. By minimizing this loss, all predictors can obtain similar target sample prediction outputs, and the final target domain sample label is obtained by averaging all outputs.

**Figure 3:**
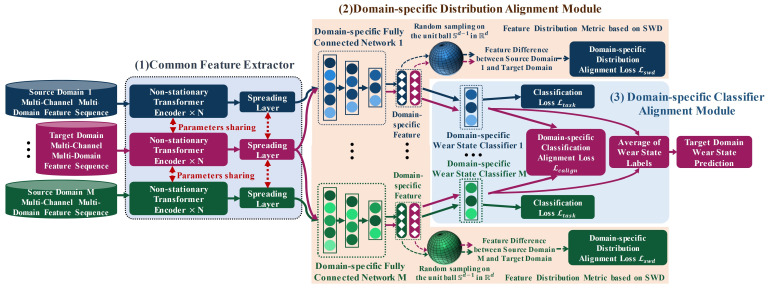
Tool wear state identification method with varying cutting parameters based on MFSAN.

Based on the two-stage alignment, this section proposes a variable-parameter tool wear state identification method based on the MFSAN. Figure 3 illustrates the overall architecture of the proposed method, consisting of one common feature extractor, one domain-specific distribution alignment module, and one domain-specific classifier alignment module. The common feature extractor is mainly composed of NSTEs and in order to avoid the consumption of computational resources caused by the repeated training of multiple networks, multi-channel multi-domain feature sequences from multiple source and target domains are mapped into the same common deep feature space by parameter sharing within the feature extractor. Furthermore, the generated common features of each pair of source domains and target domains are sent to the domain-specific distribution alignment module to mine domain-invariant representations between known cutting parameters and target cutting parameters. Meanwhile, the domain-specific feature distribution discrepancies between cutting parameters are measured based on SWD. In addition, within the domain-specific classifier alignment module, while each domain-specific wear state classifier is performing supervised training for the respective source domain samples, the module aligns the wear state probability outputs of the unlabeled samples of the target domain in the respective classifiers to obtain more reliable wear state prediction results.

### 2.3. Common Feature Extractor

**Figure 4:**
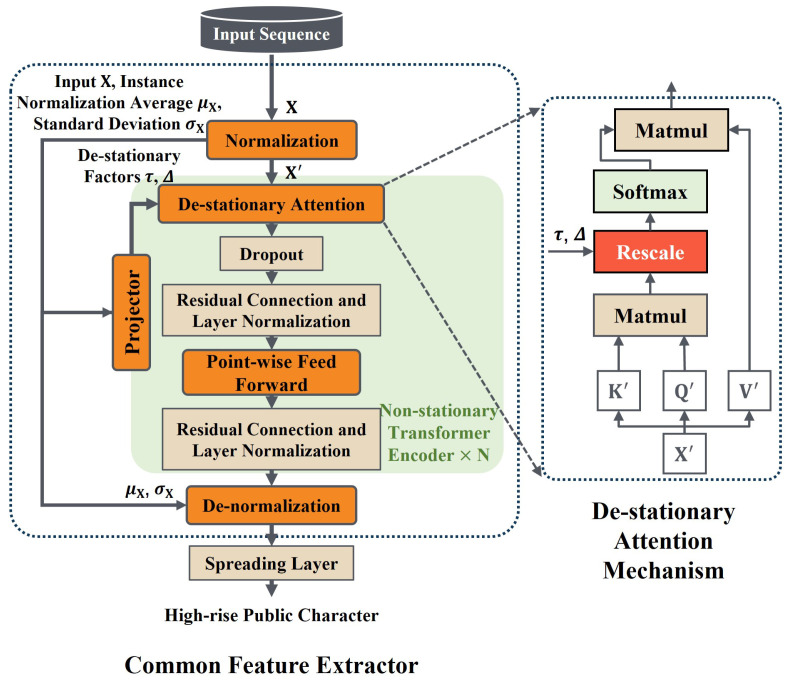
Network structure of common feature extractor network and de-stationary attention mechanism.

To accurately depict the tool wear state of milling machining, this section extracts typical features in the time domain, frequency domain, and time-frequency domain from several sensor channels and then generate the sequence of time-series features as model input. Following normalization in the preprocessing stage, the feature sequences can maintain the same scales to avoid occurrences such as gradient anomalies during model training. However, the majority of the feature sequences after this preprocessing may still exhibit non-stationary characteristics. To enhance the common feature extraction ability on these non-stationary feature sequences, this subsection applies the non-stationary Transformer encoder as the backbone network and develops the variable cutting parameter common feature extractor, as shown in Figure 4.

As shown in Figure 4, compared with the classic Transformer encoder [30], the series stationarization operation is conducted outside the NSTE [31], allowing the common feature extractor to obtain a smooth wear feature input sequence. This input sequence thus follows a stable distribution and generalizes more easily. The series stationarization operation contains instance normalization and denormalization layers. Instance normalization performs translation and scaling operations on each input sample along the temporal dimension. For a single sample $x_{i,l}=\left[x_{i,l,1},x_{i,l,},\ldots x_{i,l,l_{n}}\right]\in R^{l_{n}\times df}$ in a source domain $D_{i}^{S}=\left\{{\left(x_{i,l},y_{i,l}\right)}_{l=1}^{L}\right\}$, $l_{n}$ represents the sequence length within a single sample, and df is the feature dimension number. The instance normalization operation is as follows:

(1) $$\left\{\begin{array}{cc} \; & \mu_{x_{i,l}}=\frac{1}{l_{n}}\sum_{t=1}^{l_{n}}x_{i,l,t}\\ \; & \sigma_{x_{i,l}}^{2}=\frac{1}{l_{n}}\sum_{t=1}^{l_{n}}{\left(x_{i,l,t}-\mu_{x_{i,l}}\right)}^{2}\\ \; & x_{i,l,t}^{\prime }=\frac{1}{\sigma_{x_{i,l}}}⊙\left(x_{i,l,t}-\mu_{x_{i,l}}\right) \end{array}\right.$$

where $\mu_{x_{i,l}}$ and $\sigma_{x_{i,l}}$ represent the normalized mean and standard deviation of sample $x_{i,l}$, respectively, and their dimension is $R^{df\times 1}$. $x_{i,l,t}^{\prime }$ represents a single timestep sample after normalization. Additionally, $1/\sigma_{x_{i,l}}$ and ⊙ represent matrix element division and multiplication, respectively.

After instance normalization, the distribution of sample $x_{i,l}^{\prime }$ is more stable. After the NSTE mapping $f_{NSTE}\left(x_{i,l}^{\prime }\right)$, the encoder output enters the de-normalization layer for reverse-scale transformation to restore the lost distribution information and enters the domain-specific distribution alignment module to obtain domain-invariant representation. The de-normalization layer operates as follows:

(2) $$\left\{\begin{array}{cc} \; & z_{i,l}^{\prime }=f_{NSTE}\left(x_{i,l}^{\prime }\right)\\ \; & z_{i,l}=\sigma_{x_{i,l}}⊙\left(z_{i,l}^{\prime }+\mu_{x_{i,l}}\right) \end{array}\right.$$

where $z_{i,l}^{\prime }$ is NSTE output and $z_{i,l}$ is the reverse normalized output.

The series stationarization operation produces a more stable distribution of the encoder input. However, the scaling dot product self-attention mechanism inside the Transformer encoder is prone to over-stationarization when it performs global temporal correlation on stabilized inputs. For example, certain statistical feature sequences, such as the mean feature sequence, exhibit monotonicity along the time dimension, similar to the tool wear trend. After instance normalization during model training, these statistical feature sequences are segmented and normalized into several sequence segments with the same mean and variance, which follow more similar distributions than the sequences before stationarization. When these sequence segments enter the attention module for global temporal correlation computation, the scaled dot product self-attention mechanism may fail to recognize the monotonicity associated with wear trends, which weakens the ability of high-level feature extraction that contributes to the identification of wear states. To this end, the scaled dot product self-attention mechanism is revised into a de-stationary self-attention mechanism inside the non-stationary encoder to approximate the un-stationary attention feature map, thereby mining the non-stationary temporal dependencies related to tool wear.

Based on the assumption of linear properties and the translation invariance of the Softmax function, in order to simplify the expression, the calculation of the Softmax function of the unstationarized feature sequence input $x_{i,l}$ by the scaled dot product attention mechanism can be modified as follows [31]:

(3) $$\mathrm{Softmax}\left(\frac{{\mathrm{QK}}^{⊤}}{\sqrt{d_{k}}}\right)=\mathrm{Softmax}\left(\frac{\sigma_{x_{i,l}}^{2}Q^{\prime }K^{\prime ⊤}+1\mu_{Q}^{⊤}K^{⊤}}{\sqrt{d_{k}}}\right)$$

where Q and K represent the query matrix and key matrix corresponding to the unstationarized feature sequence input $x_{i,l}$, respectively. $d_{k}$ represents the characteristic dimension of the key matrix, and $d_{k}$ is the same as df. $Q^{\prime }$ and $K^{\prime }$ represent the query matrix and key matrix corresponding to the stationarized feature sequence input $x_{i,l}^{\prime }$, respectively. Moreover, $\sigma_{x}$ represents the instance normalized standard deviation approximation scalar and $\mu_{Q}$ represents the mean value of the query matrix Q along the time series direction with dimension $R^{d_{k}\times 1}$. 1 is an all-1 vector with dimension $R^{l_{n}\times 1}$.

Furthermore, the de-stationary factor positive scaling scalar $\tau =\sigma_{x_{i,l}}^{2}\in R^{+}$ and shifting vector $\Delta =K\mu_{Q}\in R^{l_{n}\times 1}$ are defined in the de-stationary attention mechanism. In order to effectively learn the de-stationarity factor during the training process, a multilayer perceptron (MLP) is applied as a mapper to obtain information from the statistical values $\mu_{x_{i,l}}$ and $\sigma_{x_{i,l}}$ in the unstationarized feature sequence $x_{i,l}$ and its instance normalization, respectively. The de-stationary attention mechanism can be expressed as follows:

(4) $$\left\{\begin{array}{cc} \; & \log\tau =\mathrm{MLP}\left(\sigma_{x_{i,l}},x_{i,l}\right)\\ \; & \Delta =\mathrm{MLP}\left(\mu_{x_{i,l}},x_{i,l}\right)\\ \; & \mathrm{Attn}\left(Q^{\prime },K^{\prime },V^{\prime },\tau ,\Delta \right)=\mathrm{Softmax}\left(\frac{\tau Q^{\prime }K^{\prime ⊤}+1\Delta^{⊤}}{\sqrt{d_{k}}}\right)V^{\prime } \end{array}\right.$$

where $V^{\prime }$ represents the value matrix corresponding to the stabilized feature sequence input $x_{i,j}^{\prime }$.

In the common feature extractor, the series stationarization operation and the NSTE improve the non-stationary input predictability of feature sequences and fully exploit the non-stationary timing dependencies related to tool wear. Finally, the flattening layer outputs high-level wear-related representations of each cutting parameter in the common feature space.

### 2.4. Domain-Specific Distribution Alignment Module

The domain-specific distribution alignment module sends each pair of common features from the source and target domains to domain-specific fully connected networks $F_{d_{i}}$ to extract and align domain-specific features. This module alleviates the challenge of directly aligning multiple cutting parameter distributions. The parameters are not shared among $F_{d_{i}}$, and $F_{d_{i}}$ maps common features of the target domain to obtain multiple domain-specific features. In order to mine the domain-invariant representations between the known cutting parameters and the target cutting parameters in the specific feature space and to shrink the discrepancy of their distributions in each specific feature space, this section explicitly measures the features based on the sliced-Wasserstein distance.

Wasserstein distance (WD) can mine the geometric relationships within the latent feature space and offer meaningful metrics when measuring discrepancies in feature distributions with little or no overlap. Furthermore, WD can avoid the vanishing gradient problem and reduce mode collapse during training [32]. Therefore, WD is widely used in loss function design [33] and domain adaptation research [27,34]. WD is defined as follows: Define the L2 norm as $∥\cdot ∥$. For any p≥1, the set of Borel probability measures with *p*-order moments defined on the metric space $\left(R^{d},∥\cdot ∥\right)$ with a given dimension *d* is defined as $P_{p}\left(R^{d}\right)$. For any probability measure μ,υ defined on $Z_{1},Z_{2}\subseteq R^{d}$, its probability density functions are $I_{\mu }$ and $I_{\upsilon }$, respectively, and the *p*-order WD of μ and υ are as follows:

(5) $$WD_{p}:={\left(\underset{\pi \in \prod \left(\mu ,\upsilon \right)}{\inf}\int_{Z_{1}\times Z_{2}}{∥z_{1}-z_{2}∥}^{p}d\pi \left(z_{1}-z_{2}\right)\right)}^{\frac{1}{p}}$$

where $\prod \left(\mu ,\upsilon \right)$ represents a set of transportation plans π, and the marginal distributions of π are μ and υ, respectively.

Directly using WD in deep learning scenarios will bring high computational and storage complexity [35,36]. To reduce complexity, Bonnnel et al. proposed [37,38] the SWD, a metric derived from the two ideas of an optimal transport closed-form expression for two distributions in one-dimensional space and the approach of transforming the distribution into a set of projected one-dimensional distributions using the Radon transform. $S^{d-1}$ represents the *d*-dimensional unit ball in the L2 norm for any dimension $d\in \left[2,+\infty \right)$. SWD can uniformly sample the projection direction on the unit sphere in the data ambient space and obtain the expectation of the resulting one-dimensional optimal transmission distance [33]. In order to facilitate calculation, the Monte Carlo method is usually used to extract *N* uniform sampling projection directions ${\left\{\theta_{j}\right\}}_{j=1}^{N}$ from $S^{d-1}$ for approximation:

(6) $$SWD_{p}^{p}\left(\mu ,\upsilon \right)\approx \frac{1}{N}\sum_{i=1}^{N}WD_{p}^{p}\left(ℜI_{\mu }\left(\cdot ,\theta_{j}\right),ℜI_{\upsilon }\left(\cdot ,\theta_{j}\right)\right)$$

where $ℜI_{\mu }\left(\cdot ,\theta_{j}\right)$ and $ℜI_{\upsilon }\left(\cdot ,\theta_{j}\right)$ represent Radon transform functions.

By calculating the above formula, SWD achieves lower computational cost and better scalability when calculating the discrepancy between two distributions, especially in high-dimensional statistical inference scenarios, such as measuring the distribution discrepancy in the latent feature space. Thus, this section uses 2nd-order SWD to measure the domain-specific feature distribution of each pair of source domain and target domain and obtains the average measurement value as the domain-specific distribution alignment loss $L_{\mathrm{swd}}$:

(7) $$L_{\mathrm{swd}}=\frac{1}{M}\sum_{i=1}^{M}SWD_{2}^{2}\left(F_{d_{i}}\left(F_{1}\left(x_{i}\right)\right),F_{d_{i}}\left(F_{1}\left(u\right)\right)\right)$$

By minimizing $L_{\mathrm{swd}}$ during the training process, the feature distributions between each pair of known cutting parameters and target cutting parameters are aligned, and each domain-specific fully connected network obtains the corresponding domain-invariant representation.

### 2.5. Domain-Specific Classifier Alignment Module

In the domain-specific classifier alignment module, the wear state recognition network is a multi-output network with non-parameter sharing composed of wear state classifiers $T={\left\{T_{d_{i}}\right\}}_{i=1}^{M}$ specific to each domain. Each domain-specific wear state classifier $T_{d_{i}}$ is a Softmax classifier network. On the one hand, $T_{d_{i}}$ receives the domain-specific domain-invariant representation of the corresponding source domain, identifies its corresponding wear state, and uses the cross-entropy loss function as the classification loss to optimize the network parameters. The overall classification loss calculation formula is as follows:

(8) $$L_{\mathrm{task}}=\sum_{i=1}^{M}E_{x∼D_{i}^{S}}L_{ce}\left(T_{d_{i}}\left(F_{d_{i}}\left(F_{1}\left(x_{i}\right)\right)\right),y_{i}\right)$$

where $L_{ce}\left(\cdot ,\cdot \right)$ represents the cross-entropy loss function.

On the other hand, $T_{d_{i}}$ simultaneously receives domain-specific invariant representations of target domain samples and predicts the corresponding tool wear state. The outputs of the specific wear state classifiers in each domain are inconsistent with those of the target domain samples. Especially when the target domain samples are close to the wear classification boundary, the output of each classifier may be significantly different. To this end, this section calculates the mutual difference between the probability outputs of the target domain sample on all classifiers. Then, we utilize the absolute value of the difference as the difference alignment loss $L_{\mathrm{calign}}$:

(9) $$L_{\mathrm{calign}}=\frac{2}{M\times \left(M-1\right)}\sum_{i^{\prime }=1}^{M-1}\sum_{i=i^{\prime }+1}^{M}E_{u∼D^{T}}\left[\left|T_{d_{i}}\left(F_{d_{i}}\left(F_{1}\left(u_{k}\right)\right)\right)-T_{d_{i^{\prime }}}\left(F_{d_{i^{\prime }}}\left(F_{1}\left(u_{k}\right)\right)\right)\right|\right]$$

By minimizing $L_{\mathrm{calign}}$, each domain-specific wear state classifier outputs similar wear state results for samples in the same target domain. Finally, the proposed method obtains the mean output value of each classifier to identify the wear state of samples in the target domain.

### 2.6. Training Procedure for the Proposed Method

Combined with the previous content, the overall loss function of proposed method based on the MFSAN is as follows:

(10) $$L_{\mathrm{total}}=L_{\mathrm{task}}+\beta_{1}L_{\mathrm{swd}}+\beta_{2}L_{\mathrm{calign}}$$

where $\beta_{1}$ and $\beta_{2}$ represent hyperparameter weights.

The hyperparameter weights $\beta_{1}$ and $\beta_{2}$ determine the importance of $L_{\mathrm{swd}}$ and $L_{\mathrm{calign}}$ to training, respectively. To balance the losses, the values of $\beta_{1}$ and $\beta_{2}$ during the training procedure are approximately changed as follows:

(11) $$\beta_{1}=\beta_{2}=\frac{1.8}{\left(1+\exp\left(-10\times \varsigma \right)\right)}-1$$

where ς represents the adaptive parameter, which increases linearly from 0 to 1 during training.

**Figure 5:**
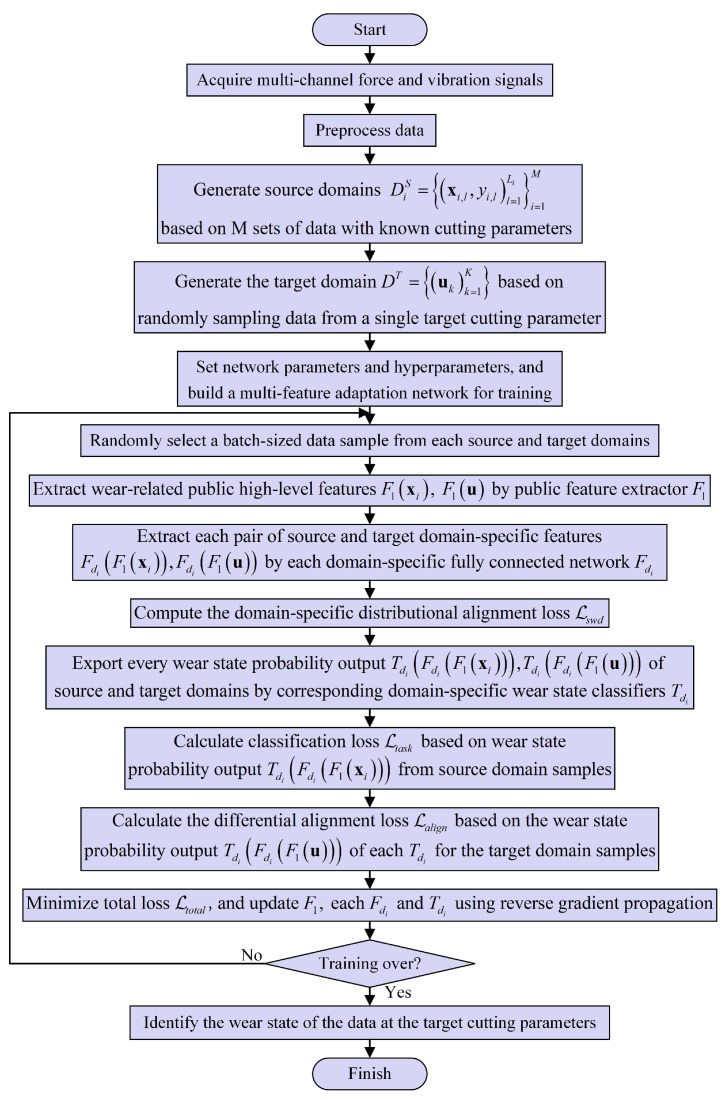
Training procedure of tool wear state identification method with varying cutting parameters based on the MFSAN .

Table 1 and Table 2 show the network parameters of the proposed method and the hyperparameters in the training stage, respectively. Figure 5 illustrates the overall training procedure of the proposed method. First, multi-channel force and vibration sensor data are collected online during machining. Data preprocessing converts high-frequency raw data into multi-channel and multi-domain statistical feature sequences. Then, the proposed method selects *M* sequence data containing known cutting parameters of the complete tool wear life cycle as the source domain input and randomly selects several unlabeled sequence data of target cutting parameters as the target domain input. Next, the training network is constructed through the settings of the above network parameters and hyperparameters. During the training procedure, each input passes through the common feature extractor, domain-specific distribution alignment module, and domain-specific classifier alignment module in sequence, and each module calculates the domain-specific distribution alignment loss $L_{\mathrm{swd}}$, classification loss $L_{\mathrm{task}}$, and output difference alignment loss $L_{\mathrm{calign}}$, respectively. Minimizing each loss function allows the network model to learn domain-specific invariant representations under each known cutting parameter and target parameter. Meanwhile, each wear state classifier improves the wear state identification accuracy of target parameter sequence data. After training, the model can accurately identify tool wear states for other sequence data within the target parameters that have not participated in the training.

## 3. Experimental Research

### 3.1. Experiment Design

**Figure 6:**
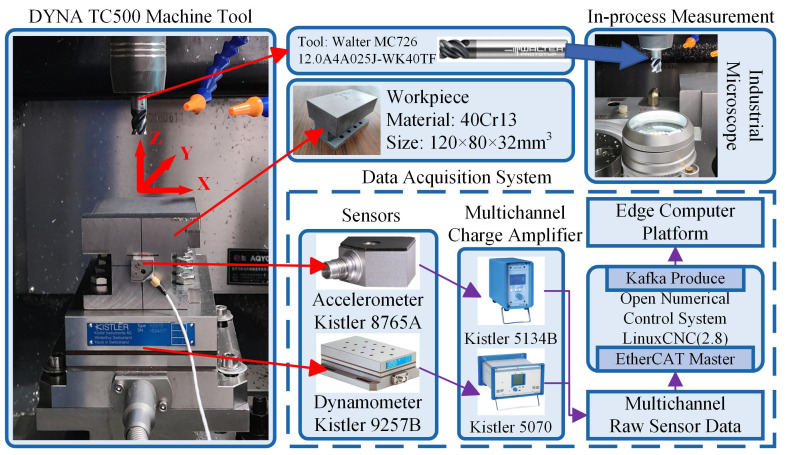
Variable cutting parameters milling experimental platform.

The experimental platform for variable cutting parameters is shown in Figure 6, and the cutting process is square-shouldered climb milling along the X-axis direction on a DYNA TC500 three-axis milling machine with nine four-flute cemented carbide resharpened end mills with TiAlN coatings. The variable cutting parameters in the milling process mainly consider three factors: cutting speed $v_{c}$, radial depth of cut $a_{e}$, and axial depth of cut $a_{p}$. Through the three-factor three-level orthogonal experiment, we obtained nine groups of cutting parameters, each tool corresponding to the cutting parameters shown in Table 3. The workpiece to be processed is I-shaped 40Cr13 steel with a hardness of 290 HB. A dynamometer and an accelerometer were installed between the worktables and workpiece to measure the cutting force signal and vibration signal with 10,000 Hz sample frequency, respectively. After the signal amplifier amplified the electrical signal generated by the sensor, it was transmitted to the local CNC system through the data acquisition module and EtherCAT bus. It was then transmitted to the edge through the Kafka message queue for processing and storage.

**Figure 7:**
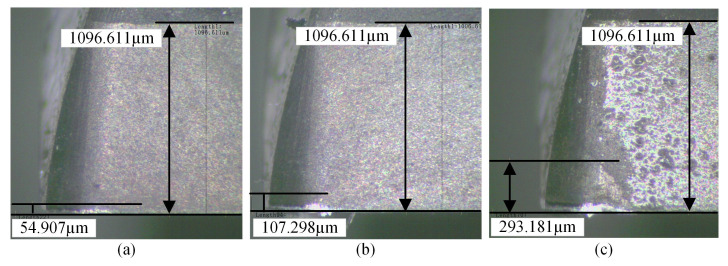
Images of flank wear at the different wear states: (**a**) slight wear (54.907 μm), (**b**) normal wear (107.298 μm), (**c**) severe wear (293.181 μm).

During the experiment, each milling stroke included four tool paths. After a certain number of milling strokes, an industrial microscope is used to measure the flank wear of the four cutting edges of the milling cutter. The maximum flank wear value is used as the wear value of the milling cutter to determine the tool wear state, including slight wear (VB ≤85μm), normal wear (85μm < VB ≤165μm), and severe wear (VB >165μm). Figure 7 shows the images of the flank surface corresponding to different tool wear states. According to the ISO 8688-1 standard [39], the tool is deemed blunt when the flank wear bandwidth reaches 300 μm.

**Figure 8:**
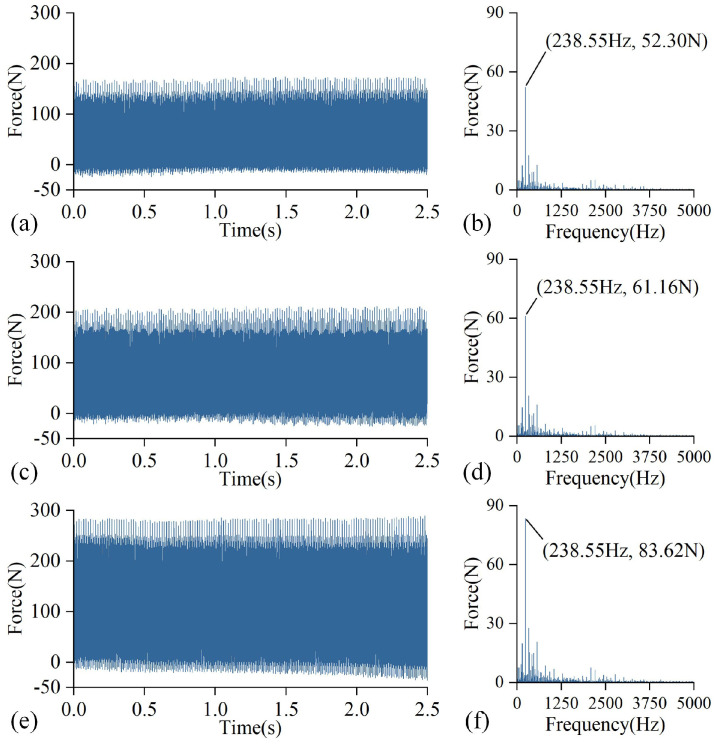
Cutting force signals in different wear states: (**a**) time domain diagram (slight wear), (**b**) frequency domain diagram (slight wear), (**c**) time domain diagram (normal wear), (**d**) frequency domain diagram (normal wear), (**e**) time domain diagram (severe wear), (**f**) frequency domain diagram (severe wear).

Figure 8 depicts the time domain and amplitude spectrum of the Y-direction milling force signal at various wear states. Using the cutting frequency of 238 Hz as an example, the amplitude of the force signal grows as tool wear increases.

### 3.2. Multi-Source Domain Unsupervised Adaptive Tasks

The raw data obtained through experiments require preliminary processing, including anomaly data removal, data segmentation, etc. After the preliminary processing of data samples, it is necessary to extract multi-dimensional statistical features, combine statistical features, and complete data normalization. First, the non-overlapping sliding window method divides the multi-channel original time series data. Then, 11 common features are extracted from each segmented data point from each sensor channel. Table 4 shows the names and mathematical expressions of the employed time, frequency, and time-frequency domain features. Statistical feature extraction is performed on each segmented data set to collect feature information about tool wear state, which can limit the impact of implicit noise during data collection. In the next step, the extracted features were stitched with the data samples to construct multi-dimensional data samples. Since the extracted statistical features have different value scales, model training is very sensitive to this. Therefore, this section uses Z-score normalization to normalize each feature along the sequence direction. Augmented Dickey-Fuller (ADF) [40] and Kwiatkowski, Phillips, Schmidt, and Shin (KPSS) [41] stationarity tests are performed on each standardized feature sequence. The results showed that many feature sequences showed non-stationary characteristics. The number of samples in each group of the obtained experimental dataset is shown in Table 5. Finally, 27 sets of multi-source domain unsupervised adaptive tasks, as shown in Table A1, are designed for model performance evaluation. For Tasks 1–9, the cutting speed parameter in the target domain does not appear in the source domain; for Tasks 10–18, the radial cutting width parameter in the target domain does not appear in the source domain; for Tasks 19–27, the axial cutting depth parameter in the target domain does not appear in the source domain. For example, in Task 1, multiple source domains are the data from cutting parameters N1–N6 ($v_{c}=$135 or 140 m/min), and the target domain is the data from cutting parameter N7 ($v_{c}$=150m/min). During the model training procedure, some unlabeled data in the target domain are randomly selected to participate in, and the remaining data are used to test the performance of the model. The results of above tasks are shown in Table A1.

### 3.3. Design of Ablation Experiment

The tool wear state identification method with variable cutting parameters proposed in this paper is based on the multi-source unsupervised domain adaptive training strategy. The NSTE builds non-stationary temporal correlations related to tool wear in the common feature extractor. Meanwhile, SWD is applied in the domain-specific distribution alignment module to measure the feature distribution difference between each pair of known cutting parameters and target cutting parameters in a specific feature space. In order to analyze and evaluate the effectiveness of each of the above key components in identifying tool wear state under varying cutting parameters, this section conducts ablation experiments on the common feature extractor network, the metric function in the domain-specific distribution alignment module, and the overall training strategy.

Firstly, in order to analyze the effectiveness of the series stationarization operation and NSTE in common feature extractor, five comparison networks (M1–M5) were designed. Compared with the proposed method, M1 retained series stationarization operation but was replaced by two classic Transformer encoders for common feature extraction. M2–M5 all removed the series stationarization operation. M2 used two classic Transformer encoders to replace the NSTE. M3 adopted a Squeeze-and-Excitation module [42]. Instead of the NSTE, M4 and M5 adopted 4-layer BiLSTM and 4-layer BiGRU networks, respectively.

Secondly, two comparison methods (M6 and M7) were designed to analyze the impact of the feature distribution discrepancy metric function in the domain-specific distribution alignment module on the wear state identification accuracy. M6 applies MMD as the metric function, a commonly used metric function in transfer learning tasks [43]. Specifically, it uses the same kernel function to map the domain-specific features under the known cutting parameters and target cutting parameters obtained in each domain-specific space to the regenerated Hilbert space to measure the discrepancy in feature distribution. M7 adopts the Correlation Alignment (CORAL) metric, which measures the covariance second-order statistical feature difference of domain-specific features under known cutting parameters and target cutting parameters to obtain the discrepancy in feature distribution [44].

Next, in order to explore the effectiveness of the multi-source unsupervised domain adaptive training strategy based on MFSAN for identifying tool wear states under varying cutting parameters, comparative methods (M8–M11) are designed to conduct ablation experiments. Among them, M8 and M9 integrate data from multiple known cutting parameters into a single training set, treat the target cutting parameter data in each group of tasks as a test set, and then adopt the supervised training strategy. M8 uses a method that combines Transformer and LSTM [45]. Furthermore, the training strategies in M10 and M11 are single-source unsupervised domain adaptive strategies. Compared with the domain division strategy of the proposed method, M10 and M11 regard the data under multiple known cutting parameters as a single source domain and select the same target cutting parameter-unlabeled data as the target domain in the proposed method. Specifically, M10 applies the Deep Adaptation Network (DAN) [46] as an overall training strategy, and M11 uses the Deep Subdomain Adaptation Network (DSAN) [47]. The feature extraction network in each comparison method is the same as the common feature extractor network of the proposed method, and the classifiers are fully connected networks with PReLU as the activation function (dimension: 792-128-64-32-3).

Finally, the methods M1–11 were employed to conduct the 27 sets of tool wear state identification tasks as outlined in Table A1. By comparing the accuracy rates of M1–11 with the proposed method in the identification tasks, an analysis and evaluation of the proposed method and the various modules used were carried out.

## 4. Analysis and Discussion

### 4.1. Results Comparison and Analysis

The accuracy of the proposed method for the 27 groups of tasks is shown in Table A1. Among them, Tasks 10–18, which perform data set segmentation based on radial cutting width, have the highest overall accuracy of 93.93%. Tasks 19–27, which perform data set segmentation based on axial cutting depth, have the lowest overall accuracy of 92.11%. The overall accuracy of Tasks 1–9, which perform data set segmentation based on cutting speed, is similar to that of Tasks 10–18, which is 93.63%. The wear state recognition accuracy of the proposed method in each task ranges from 86.01% to 98.45%, with an average accuracy of 93.22%. The accuracy of the proposed method is lower than 90% on four groups of tasks, except for Tasks 3, 12, 21, and 24, and is higher than 90% on other tasks. Overall, the proposed method can realize tool wear state identification under variable cutting parameters with high accuracy.

**Figure 9:**
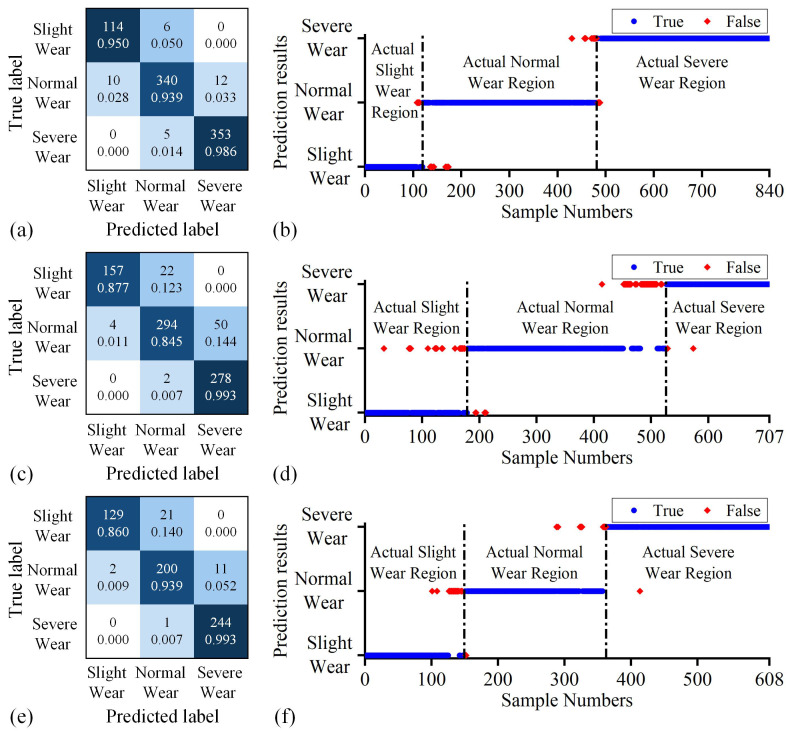
Performance of the proposed method in Tasks 7–9: (**a**) confusion matrix in Task 7, (**b**) Prediction results in Task 7, (**c**) confusion matrix in Task 8, (**d**) prediction results in Task 8, (**e**) confusion matrix in Task 9, (**f**) prediction results in Task 9. In the confusion matrix, the deeper the color, the higher the proportion it signifies.

This section adopts the confusion matrix and data prediction results to analyze and express the performance of the proposed method further. First, taking Tasks 7–9 as examples, the recognition confusion matrices and prediction results of the proposed method for target domain cutting data are depicted in Figure 9. The average recognition accuracy of the proposed method for the three sets of tasks is 93.55%. Figure 9 illustrates that the primary error source of the proposed method is the misjudgment of wear state data close to the classification boundary. In Tasks 7–9, the actual slight wear and actual normal wear data close to the classification boundary are misjudged as normal and severe wear state in advance, respectively. Among them, for Task 8, 12.29% of the actual slight wear data and 14.37% of the actual normal wear data are misjudged as normal and severe wear state in advance.

**Figure 10:**
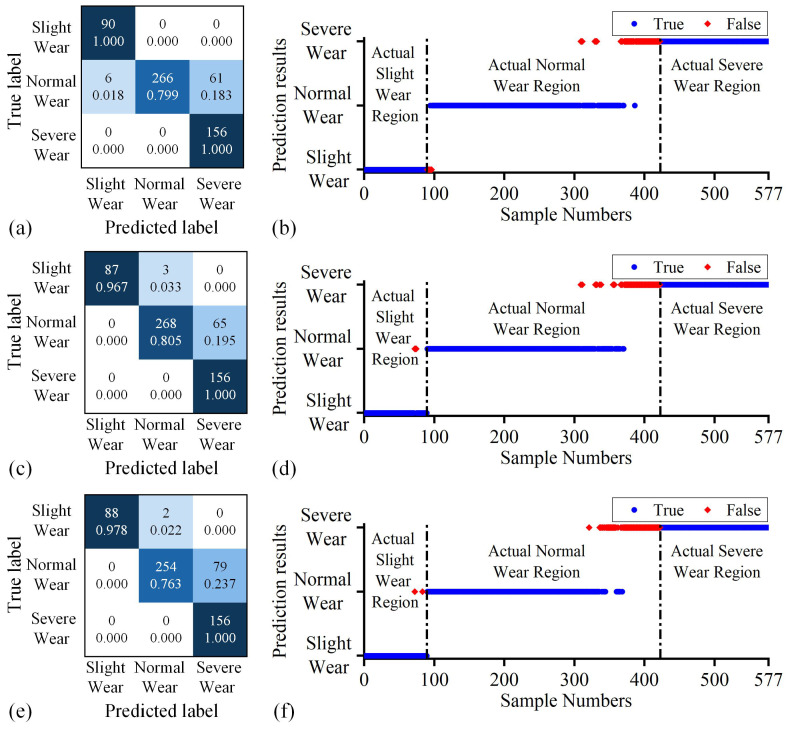
Performance of the proposed method in Tasks 3, 12, and 24: (**a**) confusion matrix in Task 3, (**b**) prediction results in Task 3, (**c**) confusion matrix in Task 12, (**d**) prediction results in Task 12, (**e**) confusion matrix in Task 24, (**f**) prediction results in Task 24. In the confusion matrix, the deeper the color, the higher the proportion it signifies.

In addition, the three tasks with the lowest recognition accuracy among the 27 tasks performed using the proposed method were further analyzed, including Task 3 (88.43%), Task 12 (88.26%), and Task 24 (86.01%). Figure 10 depicts the corresponding recognition confusion matrices and prediction results in the target domain. It is observed that the target domains of these three tasks are all data sets corresponding to N9 cutting parameters. In these three tasks, the proposed method achieved 98.15% and 100% recognition accuracy for actual slight wear samples and actual severe state data, respectively, while only achieving 78.88% recognition accuracy for actual normal wear samples. From the data prediction results in Figure 10, the main misjudgment of N9 cutting parameter data by the proposed method is that actual normal wear samples close to the classification boundary are continuously misjudged as severe wear in advance. In Tasks 3, 12, and 24, approximately 18.32%, 19.52%, and 23.72% of the actual late normal wear samples were misjudged in advance, respectively. This phenomenon may be because the workpiece in Figure 6 has a thin-walled structure after a large amount of cutting. Continuing to use this workpiece for machining under N9 cutting parameters leads to chatter. Chatter interference information was coupled with tool wear-related information, and the feature extractor captured the corresponding generated features, resulting in model identification errors. In addition, the increase in signal value caused by the larger cutting parameters of N9 may have also exacerbated this problem, and its life expectancy is also the shortest among all groups of cutting parameters. This viewpoint is supported by Table A1. Among the 27 sets of tasks, the proposed method exhibits lower recognition accuracy in Tasks 10, 18, 19, and 21, where the target domains are N3 and N7. These two datasets correspond to relatively large processing parameters: N3 has the largest $a_{e}$ and $a_{p}$, while N7 has the largest $v_{c}$ and $a_{p}$. It is worth noting that the proposed method can correctly identify 100% of the actual mid- to late-stage severe wear data in all tasks.

**Figure 11:**
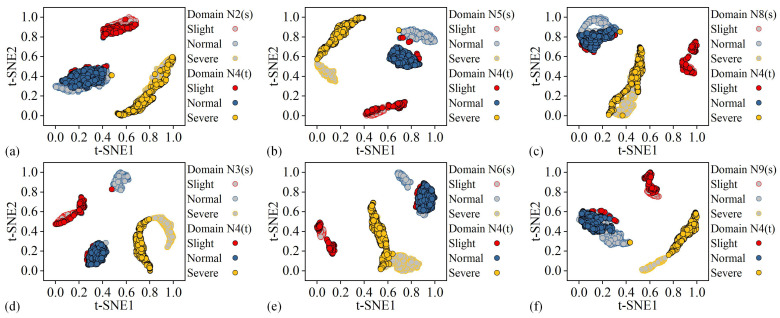
Feature visualization of each domain-specific classifier in Task 17: (**a**) N2 (source)-N4 (target), (**b**) N5 (source)-N4 (target), (**c**) N8 (source)-N4 (target), (**d**) N3 (source)-N4 (target), (**e**) N6 (source)-N4 (target), (**f**) N9 (source)-N4 (target).

**Figure 12:**
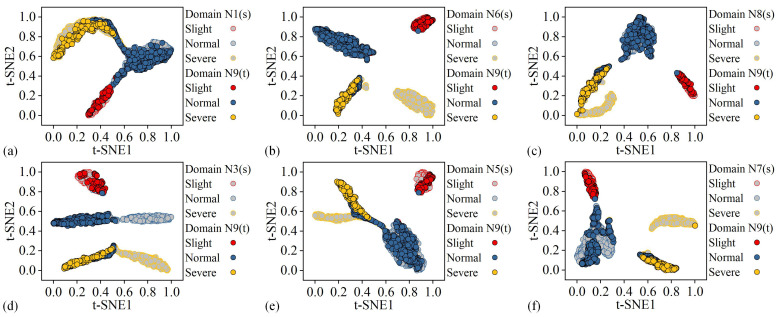
Feature visualization of each domain-specific classifier in Task 24: (**a**) N1 (source)-N9 (target), (**b**) N6 (source)-N9 (target), (**c**) N8 (source)-N9 (target), (**d**) N3 (source)-N9 (target), (**e**) N5 (source)-N9 (target), (**f**) N7 (source)-N9 (target).

Among the 27 tasks, Task 17 (98.45%), which has the highest wear state recognition accuracy, and Task 24 (86.01%), which has the lowest wear state recognition accuracy, were studied further. The t-distributed stochastic neighbor embedding (t-SNE) method was utilized to visualize the features within the domain-specific wear state classifiers of the proposed methods in the two tasks, as shown in Figure 11 and Figure 12, respectively. In the Figure 11 and Figure 12, red, blue, and yellow represent slight, normal, and severe wear states, respectively. Additionally, the samples filled with gray are the features of each known cutting parameter training data in the corresponding domain-specific classifier. Samples filled in red, blue, and yellow are the features of the target cutting parameter test data in the corresponding domain-specific classifiers.

In Figure 11, each domain-specific network can separate the data under different wear states in each pair of source and target domains. Meanwhile, the data in each pair of source domain and target domain under the same wear state achieve a better degree of mutual aggregation. In Figure 12, some of the actual normal wear state data of the target cutting parameters are aggregated with the actual severe wear data in each known parameter, consistent with the data prediction results in Figure 10c. Overall, the proposed method enables each domain-specific network to learn the domain-invariant representation between each pair of known cutting parameters and target cutting parameters through the two-stage alignment, achieving cross-domain inter-class separation of the tool wear state under variable cutting parameters.

### 4.2. Ablation Studies

**Figure 13:**
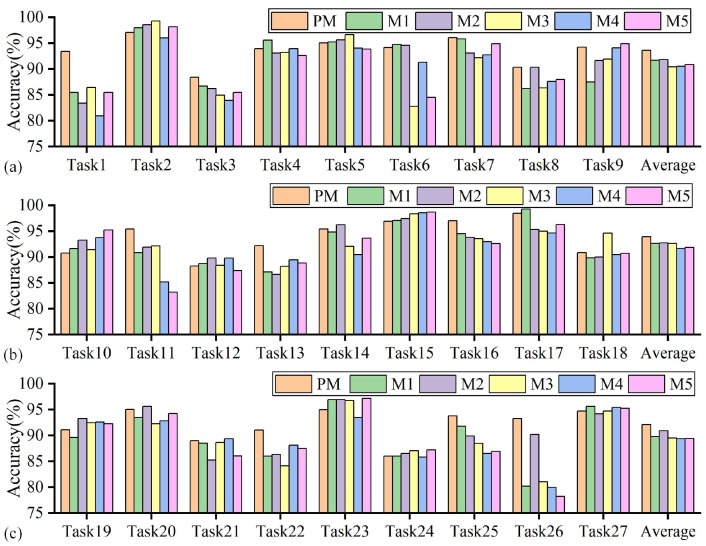
Comparison of prediction results between the proposed method and other methods (different feature extractors): (**a**) Tasks 1–9, (**b**) Tasks 10–18, (**c**) Tasks 19–27.

The accuracy of the proposed method and methods M1-5 in tool wear state identification across 27 sets of variable cutting parameters is presented in Figure 13 and Table A2. It is observed that the proposed method has the most significant number of groups with the highest accuracy on 27 tasks and achieved the highest average accuracy of 93.22% on 27 groups of tasks. Compared with the M1–M5 method, the average accuracy of the proposed method increased by 1.84%, 1.41%, 2.36%, 2.70%, and 2.50%, respectively. Second, the proposed method achieves higher recognition accuracy and has the best accuracy stability compared with the other four methods. Specifically, the highest accuracy rates of the proposed method and methods M1–M5 on each group of tasks were around 98% to 99%. In comparison, the lowest accuracy rates on each group of tasks were 86.01%, 80.17%, 83.39%, 81.05%, 79.96%, and 78.21%, respectively. The differences between the highest and lowest recognition accuracy rates were 12.44%, 19.11%, 15.15%, 18.23%, 18.59%, and 20.51%, respectively. Third, when combining the proposed method with methods M1–3, it was found that using only the series stationarization operation on the Transformer encoder network architecture would reduce performance. This phenomenon may be due to the performance degradation of the scaled-dot product attention mechanism in identifying non-stationary temporal features related to wear trends after over-stationarity. The series stationarization operation and the de-stationary attention mechanism serve as complementary modules and jointly participate in extracting common wear features to enhance accuracy. In addition, comparing methods PM, M4, and M5, it was found that the performance of the common feature extractor based on the Transformer encoder architecture was improved, indicating that the wear-related global temporal correlation constructed by it is more conducive to improving the accuracy of wear state identification.

**Figure 14:**
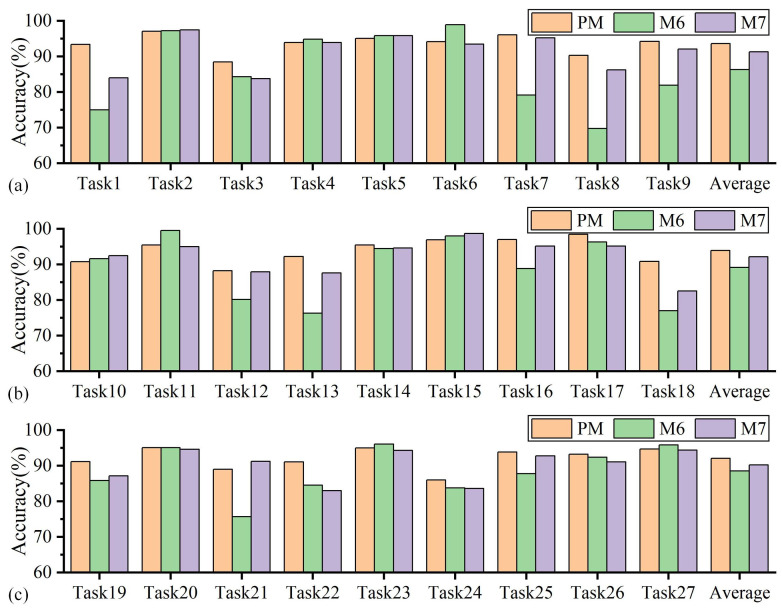
Comparison of prediction results between the proposed method and other methods (different domain-specific feature metric functions): (**a**) Tasks 1–9, (**b**) Tasks 10–18, (**c**) Tasks 19–27.

The accuracy of the proposed method and methods M6–7 in tool wear state identification across 27 sets of variable cutting parameters is presented in Figure 14 and Table A2. First, as shown in Figure 14, the proposed method achieved the highest accuracy in 17 wear state identification tasks, compared with M6 (7 tasks) and M7 (5 tasks). The average accuracy rates of these three methods on each group of tasks were 93.22%, 88.00%, and 91.23%, respectively. Second, compared with the other two methods, the SWD feature distribution discrepancy measure can achieve higher recognition accuracy. Meanwhile, the stability of the wear state recognition accuracy was also the best. Specifically, the highest accuracy rates of the proposed method, M6, and M7, for each group of tasks were all over 98%. The lowest accuracy rates on each group of tasks were 86.01%, 69.77%, and 82.54%, respectively. The differences between the highest and lowest recognition accuracy rates were 12.44%, 29.80%, and 16.19%, respectively. The above results show the effectiveness of utilizing SWD as a metric function to align domain-specific distributions and learn domain-specific domain-invariant representations. Thus, SWD can help improve identification accuracy when used in tool wear state identification tasks under variable cutting parameters.

**Figure 15:**
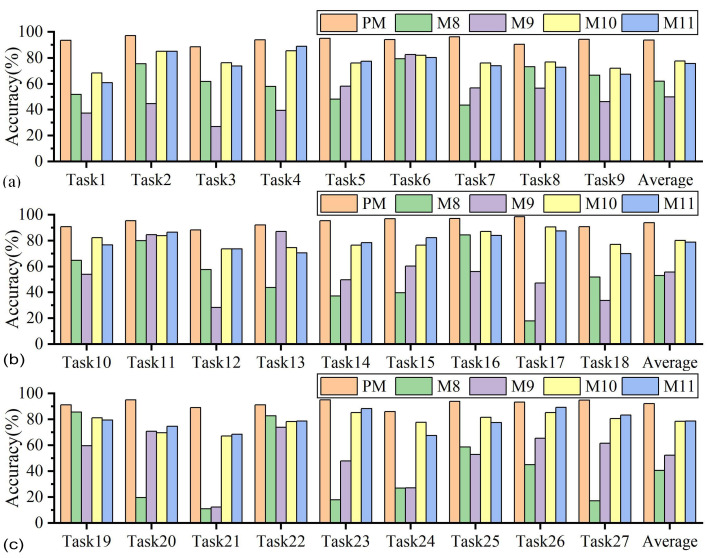
Comparison of prediction results between the proposed method and other methods (different training strategies): (**a**) Tasks 1–9, (**b**) Tasks 10–18, (**c**) Tasks 19–27.

The accuracy of the proposed method and methods M8–11 in tool wear state identification across 27 sets of variable cutting parameters is presented in Figure 15 and Table A2. As depicted in Figure 15, the accuracy of M8 and M9 was notably worse than that of the other three methods, with an average accuracy of only 51.87% (M8) and 52.67% (M9) across 27 tasks. This result indicates that relying solely on a supervised training strategy for identifying tool wear states under varying cutting parameters is insufficient to fulfill the few-shot scenario requirements. Due to the alteration in the known and target cutting parameters, there is a notable discrepancy in the data feature distributions. Consequently, the supervised training strategy can lead to the network model overfitting.

The average accuracy rates of M10 and M11 on the 27 tasks were 78.78% and 77.70%, respectively, higher than M9 (52.67%). However, compared with the proposed method, their average accuracy dropped by 14.44% and 15.52%, respectively. In addition, compared with M8–M11, the proposed method achieved the best wear state identification accuracy in all 27 groups of tasks. It can be inferred that it is difficult to integrate multiple known cutting parameter data into a source domain and to learn the common domain invariant representation of multiple known cutting parameter data and target cutting parameter data in the common feature space. Thus, under the single-source unsupervised adaptive training strategy, subtle feature differences between each cutting parameter may not be recognized, impairing the wear state identification performance under variable cutting parameters.

## 5. Conclusions and Future Works

A novel wear state identification method with variable cutting parameters based on the multi-source unsupervised adaptive training strategy is proposed. The performance and effectiveness of the proposed method were evaluated and analyzed. The main conclusions are as follows:

(1) A multi-source unsupervised domain adaptive training strategy based on MFSAN boosts tool wear state identification accuracy under variable cutting parameter scenarios. The strategy fully utilizes multiple known cutting parameter data sets and effectively achieves mutual separation of wear states under varied cutting parameters by aligning domain-specific feature distribution and domain-specific classifier output in two stages.
(2) The common feature extractor based on the NSTE and the domain-specific feature distribution measure with SWD assist in improving the wear state classification performance.
(3) The effectiveness of the proposed method is evaluated through the tasks of identifying tool wear status with variable cutting parameters. Among 27 sets of tasks, the proposed method demonstrates an average accuracy of 93.22%, representing a significant enhancement of 14.44% over methods such as DAN and DSAN. The use of NSTE and SWD improves the recognition accuracy of the proposed method by 1.41% and 1.99%, respectively.

Although this paper has studied the implementation of tool wear identification under variable cutting parameter conditions, it still can be improved. Further studies could be conducted on the generalization of wear monitoring tasks under complex variable working conditions, like variable processing paths and cross-processing equipment, or on interpretable models for simultaneous tool wear and breakage detection. 

## Figures and Tables

**Table 1:** Network model parameters.

Network Modules	Parameters
NSTE	Number of encoders: 2, non-stationary self-attention head number: 1
Point-wise Feed Forward	Convolution kernel size of one-dimensional convolutional layer 1:1, padding: 0, input channel dimension: 66, output channel dimension: 264; convolution kernel size of one-dimensional convolutional layer 2:1, padding: 0, input channel dimension: 264, output channel dimension: 64
Projector	Number of hidden layers: 1, dimension of hidden layer: 64, activation function: ReLU
Domain-specific fully connected network	Dimensions of each hidden layer: 792-128-64-32, activation function: PReLU
Domain-specific classifer	Dimensions of hidden layer: 32-3

**Table 2:** Hyperparameters for the proposed model training.

Hyperparameters	Value	Hyperparameters	Value
Batch size	32	Optimizer	AdamW
Training times	100	Weight decay in the optimizer	0.00005
Learning rate (LR)	0.0008	Momentum in the optimizer	0.9
LR scheduler	Cosine Annealing Warm Up	Dropout	0.1
LR warmup steps	15	Number of SWD projection directions	320

**Table 3:** Milling experimental cutting parameters.

No.	$v_{c}$ (m/min)	$a_{e}$ (mm)	$a_{p}$ (mm)	$f_{z}$ (mm/r)	*n* (rpm)
N1	135	1.5	0.6	0.116	3580
N2	135	2	0.7	0.116	3580
N3	135	2.5	0.8	0.116	3580
N4	140	1.5	0.7	0.116	3710
N5	140	2	0.8	0.116	3710
N6	140	2.5	0.6	0.116	3710
N7	150	1.5	0.8	0.116	3980
N8	150	2	0.6	0.116	3980
N9	150	2.5	0.7	0.116	3980

**Table 4:** Statistical features in time, frequency, and time–frequency domain [42].

No.	Feature	Formula
1	Mean	$x_{mean}=\frac{1}{N}\sum_{i=1}^{N}\left|x_{i}\right|$
2	Root mean square	$x_{rms}=\sqrt{\frac{1}{N}\sum_{i=1}^{N}x_{i}^{2}}$
3	Max	$x_{s}=\max\left(x_{i}\right)$
4	Standard deviation	$x_{sd}=\sqrt{\frac{1}{N}\sum_{i=1}^{N}{\left(\left|x_{i}\right|-x_{mean}\right)}^{2}}$
5	Peak value	$x_{p}=\max\left(\left|x_{i}\right|\right)$
6	Peak-to-peak	$x_{pp}=\max\left(x_{i}\right)-\min\left(x_{i}\right)$
7	Spectral power	$f_{sp}=\sum_{i=1}^{N}{\left(f_{i}\right)}^{3}P\left(f_{i}\right)$
8	Frequency centroid	$f_{fc}=\sum_{i=1}^{N}f_{i}P\left(f_{i}\right)/\sum_{i=1}^{N}P\left(f_{i}\right)\;$
9	Root mean square frequency	$f_{rmsf}=\sqrt{\sum_{i=1}^{N}f_{i}^{2}P\left(f_{i}\right)/\sum_{i=1}^{N}P\left(f_{i}\right)}$
10	Root variance frequency	$f_{rvf}=\sqrt{\sum_{i=1}^{N}{\left(f_{i}-f_{fc}\right)}^{2}P\left(f_{i}\right)/\sum_{i=1}^{N}P\left(f_{i}\right)}$
11	Wavelet packet energy	$e_{wpe}=\sum_{i=1}^{N}wt_{\varphi }^{2}\left(i\right)/N\;$

**Table 5:** Sample numbers for each group in the experimental dataset.

No.	Number of Slight Wear Samples	Number of Normal Wear Samples	Number of Severe Wear Samples	Total Number of Samples
N1	80	235	248	563
N2	144	272	252	668
N3	96	144	192	432
N4	100	200	304	604
N5	72	192	156	420
N6	44	88	200	332
N7	60	280	224	564
N8	72	164	216	452
N9	60	239	128	427

## Data Availability

The data that support the findings of this study are available from the corresponding author upon reasonable request.

## Appendix A

**Table A1:** Task division and the wear state recognition accuracy of the proposed method in each task.

No.	Source Domain	Target Domain	Accuracy (%)	Average Accuracy (%)	Overall Accuracy (%)
Task1	N1, N2, N3, N4, N5, N6	N7	93.41	92.97	93.63
Task2	N1, N2, N3, N4, N5, N6	N8	97.09		
Task3	N1, N2, N3, N4, N5, N6	N9	88.43		
Task4	N1, N2, N3, N7, N8, N9	N4	93.93	94.36	
Task5	N1, N2, N3, N7, N8, N9	N5	95.04		
Task6	N1, N2, N3, N7, N8, N9	N6	94.12		
Task7	N4, N5, N6, N7, N8, N9	N1	96.07	93.55	
Task8	N4, N5, N6, N7, N8, N9	N2	90.34		
Task9	N4, N5, N6, N7, N8, N9	N3	94.24		
Task10	N1, N4, N7, N2, N5, N8	N3	90.79	91.49	93.93
Task11	N1, N4, N7, N2, N5, N8	N6	95.43		
Task12	N1, N4, N7, N2, N5, N8	N9	88.26		
Task13	N1, N4, N7, N3, N6, N9	N2	92.19	94.84	
Task14	N1, N4, N7, N3, N6, N9	N5	95.44		
Task15	N1, N4, N7, N3, N6, N9	N8	96.91		
Task16	N2, N5, N8, N3, N6, N9	N1	97.02	95.44	
Task17	N2, N5, N8, N3, N6, N9	N4	98.45		
Task18	N2, N5, N8, N3, N6, N9	N7	90.84		
Task19	N1, N6, N8, N2, N4, N9	N3	91.12	91.72	92.11
Task20	N1, N6, N8, N2, N4, N9	N5	95.04		
Task21	N1, N6, N8, N2, N4, N9	N7	89.01		
Task22	N1, N6, N8, N3, N5, N7	N2	91.08	90.70	
Task23	N1, N6, N8, N3, N5, N7	N4	95.00		
Task24	N1, N6, N8, N3, N5, N7	N9	86.01		
Task25	N2, N4, N9, N3, N5, N7	N1	93.81	93.92	
Task26	N2, N4, N9, N3, N5, N7	N6	93.25		
Task27	N2, N4, N9, N3, N5, N7	N8	94.72		

**Table A2:** Results of ablation experiment.

No.	Accuracy (%)											
	PM	M1	M2	M3	M4	M5	M6	M7	M8	M9	M10	M11
Task1	93.41	85.47	83.39	86.45	80.95	85.47	74.97	84.01	51.84	37.36	68.50	61.05
Task2	97.09	98.00	98.54	99.27	95.99	98.18	97.27	97.45	75.57	44.81	85.06	85.06
Task3	88.43	86.70	86.18	84.97	83.94	85.49	84.28	83.77	61.98	26.98	76.34	73.92
Task4	93.93	95.60	93.10	93.21	93.93	92.62	94.88	93.93	58.13	39.38	85.36	88.93
Task5	95.04	95.24	95.64	96.63	94.05	93.85	95.83	95.83	48.21	58.33	76.19	77.58
Task6	94.12	94.77	94.55	82.79	91.29	84.53	98.91	93.46	79.44	82.52	81.92	80.39
Task7	96.07	95.83	93.10	92.14	92.74	94.88	79.17	95.24	43.75	57.02	76.07	74.05
Task8	90.34	86.25	90.34	86.37	87.61	87.98	69.77	86.25	73.30	56.69	76.95	72.86
Task9	94.24	87.50	91.61	91.94	94.08	94.90	81.91	92.11	66.75	46.36	72.20	67.43
Task10	90.79	91.61	93.26	91.45	93.75	95.23	91.61	92.43	64.75	54.13	82.24	76.65
Task11	95.43	90.85	91.94	92.16	85.19	83.22	99.56	94.99	79.93	84.64	83.88	86.49
Task12	88.26	88.77	89.81	88.43	89.81	87.39	80.14	87.91	57.68	28.28	73.58	73.58
Task13	92.19	87.11	86.62	88.23	89.47	88.85	76.33	87.61	43.85	87.08	74.60	70.51
Task14	95.44	94.84	96.23	92.06	90.48	93.65	94.44	94.64	37.20	49.85	76.59	78.37
Task15	96.90	97.09	97.45	98.36	98.54	98.73	98.00	98.73	39.63	60.38	76.50	82.33
Task16	97.02	94.52	93.81	93.57	92.98	92.62	88.81	95.12	84.38	56.21	87.02	84.05
Task17	98.45	99.29	95.36	95.00	94.64	96.31	96.31	95.12	17.86	47.23	90.60	87.50
Task18	90.84	89.87	89.99	94.63	90.48	90.72	77.05	82.54	51.84	33.61	77.17	70.09
Task19	91.12	89.64	93.26	92.43	92.60	92.27	85.86	87.17	85.63	59.56	81.09	79.44
Task20	95.04	93.45	95.64	92.26	92.86	94.25	95.04	94.64	19.64	70.83	69.64	74.60
Task21	89.01	88.52	85.23	88.65	89.38	86.08	75.70	91.21	11.03	12.27	67.16	68.50
Task22	91.08	86.00	86.37	84.14	88.10	87.49	84.51	83.02	82.67	73.79	78.32	78.69
Task23	95.00	96.91	96.91	96.79	93.45	97.14	96.07	94.29	17.86	47.86	85.12	88.21
Task24	86.01	86.01	86.53	87.05	85.84	87.22	83.77	83.59	26.95	27.11	77.72	67.53
Task25	93.81	91.79	89.88	88.45	86.55	86.91	87.74	92.74	58.64	52.90	81.55	77.50
Task26	93.25	80.17	90.20	81.05	79.96	78.21	92.38	91.07	45.07	65.36	85.19	89.33
Task27	94.72	95.63	94.17	94.72	95.45	95.26	95.81	94.35	17.05	61.61	80.51	83.24
Average	93.22	91.39	91.82	90.86	90.52	90.72	88.00	91.23	51.87	52.67	78.78	77.70
